# Aurora kinase inhibitors: Potential molecular-targeted drugs for gynecologic malignant tumors (Review)

**DOI:** 10.3892/br.2019.1249

**Published:** 2019-11-01

**Authors:** Kiyoko Umene, Kouji Banno, Iori Kisu, Megumi Yanokura, Yuya Nogami, Kosuke Tsuji, Kenta Masuda, Arisa Ueki, Yusuke Kobayashi, Wataru Yamagami, Hiroyuki Nomura, Eiichiro Tominaga, Nobuyuki Susumu, Daisuke Aoki

Biomed Rep 1:335-340, 2013; DOI: 10.3892/br.2013.91

Subsequently to the publication of the above review article, the authors have realized that they made an error in terms of the description of the Aurora kinase inhibitor, ZM44739. Concerning the sentence in the section ‘3. Clinical applications of Aurora kinase inhibitors’, on p. 337, right-hand column, lines 7-8, the authors wrote ‘ZM447439 was reported as the first Aurora kinase inhibitor in 2003 and is currently in phase I clinical trials…’; however, a phase I clinical trial with this drug has not yet been conducted. Therefore, this sentence should be corrected to the following: ‘ZM447439 was reported as the first Aurora kinase inhibitor in 2003’.

The authors apologize to the readership of the Journal for the misinformation in this regard, and for any inconvenience caused.

